# Operative timing and surgical complexity in hand trauma: a multicenter analysis of replantation and non-replantation centers in Germany

**DOI:** 10.1007/s00402-026-06363-8

**Published:** 2026-05-29

**Authors:** Antek Nicklas, Patrick Will Marks, Adrian Dragu, Hannah Schmidt, Andre Rotärmel, HandTraumaRegister DGH

**Affiliations:** 1 University Center for Orthopedics, Trauma and Plastic Surgery, Faculty of Medicine and University Hospital Carl Gustav Carus, TU Dresden, Dresden, Germany; 2 Academy for Trauma Surgery (AUC), Munich, Germany; 3 Working Group HandTraumaRegister of the German Society for Hand Surgery (AG HTR DGH), Munich, Germany

**Keywords:** Amputation, Hand surgery, HandTraumaRegister, Flexor tendon injury, Extensor tendon injury

## Abstract

**Background:**

Hand injuries are common traumatic conditions that often require specialized surgical care. Differences in hospital structures and levels of specialization may influence the timeliness and complexity of treatment. This study evaluates quality of care across different hospital types, focusing on time-to-skin-incision and surgical management of finger and hand injuries.

**Materials and methods:**

This retrospective multicenter study is based on data from the HandTraumaRegister of the German Society for Hand Surgery (HTR DGH). A total of 16,726 surgically treated cases with documented finger or hand injuries recorded between 2018 and 2023 were analyzed. Hospitals were categorized as non-replantation centers, replantation centers, or FESSH-accredited replantation centers. Demographic characteristics, injury-patterns, treatment modalities, and time-related parameters were assessed. Statistical analysis included descriptive statistics, group comparisons, and multivariate regression models to evaluate factors associated with time-to-skin-incision.

**Results:**

The study population consisted predominantly of male patients (75.79%), and 91.6% were right-handed. Soft tissue injuries without fractures or amputations accounted for 46.57% of cases. Non-replantation centers demonstrated the longest time-to-skin-incision (median 13 h), whereas replantation centers (median 3.75 h) and FESSH-accredited centers (median 4 h) showed significantly shorter times. Treatment strategies differed significantly between center types (*p* < 0.001), with non-replantation centers performing more secondary procedures. Replantations or revascularizations were performed in 3.96% of cases, with the highest proportion observed in FESSH-accredited centers (4.64%). Operative durations were significantly longer in replantation centers, particularly for complex and amputation injuries (*p* < 0.001).

**Conclusion:**

Specialized replantation centers are associated with shorter access times to surgical care compared to non-replantation centers. Longer operative times observed in highly specialized and FESSH-accredited centers may reflect greater injury complexity as well as the technical demands of advanced microsurgical procedures, rather than indicating differences in care quality. These findings highlight the potential relevance of specialized care structures in the management of severe finger injuries.

## Introduction

 Hand injuries are among the most common injuries requiring surgical intervention and encompass a broad spectrum, ranging from simple soft tissue injuries to complex, multiphalangeal damage [1]. A study by Crowe and colleagues demonstrated that the global incidence of hand injuries has only slightly decreased since 1990. In 2017, the age-standardized incidence of hand and wrist fractures was 179 per 100,000 population [2].

Severe hand injuries, such as amputations or combined injuries involving bones, tendons, nerves, and vessels, not only result in significant functional impairment for patients but also pose a considerable challenge to the healthcare system. For example, de Putter and colleagues showed that the costs of hand and wrist injuries in the Netherlands amount to approximately 740 million US dollars per year, and that hand and wrist injuries account for about 20% of emergency department visits [3].

Similarly, Sears and colleagues demonstrated a favorable cost-utility ratio for replantations compared to amputations in terms of quality-adjusted life years (QALY) [4]. In their 2014 study, they found that especially the replantation of three or four fingers was associated with relatively low cost-utility ratios (27,100 and 23,800 US dollars per quality-adjusted life year, respectively) [4]. Furthermore, replantation for distal thumb amputations showed a relatively low incremental cost-utility ratio compared to replantation of non-thumb-related distal amputations [4].

Adequate management of these injuries often requires highly specialized microsurgical expertise, interdisciplinary collaboration, and access to advanced infrastructure.

Against the background of the current hospital reform in Germany, which aims for greater centralization and specialization of medical care, the importance of specialized care centers becomes particularly evident. The reform seeks to improve the quality of care by transferring complex cases to highly specialized facilities. Consequently, for hand surgery, especially in cases involving multiple functional structures, this development presents an opportunity to implement standardized treatment protocols, optimized processes, and a higher level of patient safety particularly in complex hand injuries with several functional structures damaged. Osterhoff and colleagues have already conducted initial studies on the quality of care in the context of the upcoming reform, using proximal femoral fractures as an example [5].

**Fig. 1:**
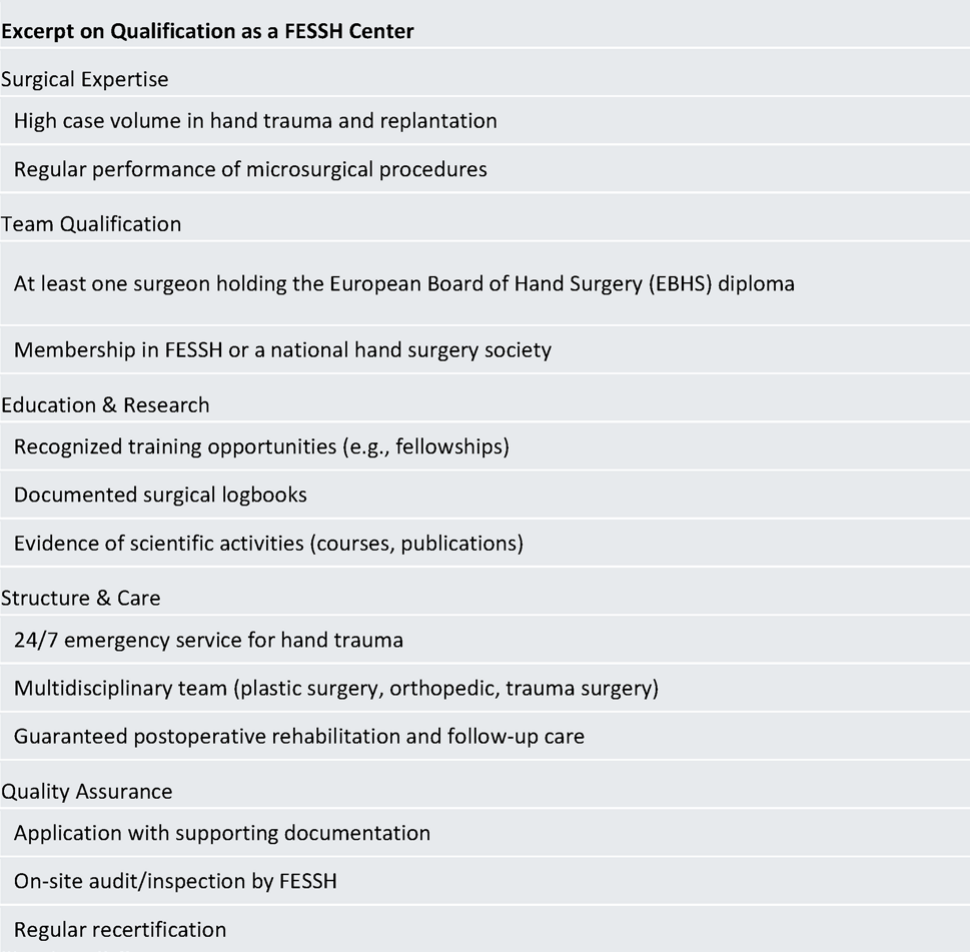
Excerpt on Qualification as a FESSH Center

Specialized replantation centers, as well as centers certified by the Federation of European Societies for Surgery of the Hand (FESSH), play a central role in this context, as their structure and expertise enable them to treat even complex cases promptly and at the highest medical standards. To obtain FESSH certification, a hospital must demonstrate that it meets high standards in hand surgery. The criteria that a clinic must fulfill to obtain such certification are outlined in Fig. 1.

Especially with regard to the rapid and timely treatment of severe injuries such as replantations following amputation injuries, a short door-to-skin-incision time is considered crucial for optimal patient management. In this context, a meta-analysis on replantation published in 2024 did not demonstrate a significant influence of ischemia time on replantation outcomes, thereby suggesting that other time-related and organizational factors may play a more relevant role in determining overall results [6].

Preliminary evidence supports the hypothesis that targeted primary transfer to specialized centers by the first responders could potentially improve outcomes and patient satisfaction, as studies from Taiwan have shown that lower surgical expertise is associated with a higher rate of replantation failure [7]. Hsu and colleagues demonstrated that, among 13,416 finger replantations, procedures performed by trainees during their surgical training had a significantly higher failure rate compared to those performed by experienced microsurgeons (15.5% vs. 7.6%; odds ratio 2.0; confidence interval 1.1–3.7). This not only affects functional restoration but also the long-term quality of life for affected patients [8].

Against this background, the present study investigates the focus and differences in care between specialized and non-specialized centers using data from the HandTraumaRegister (HTR DGH) of the German Society for Hand Surgery (DGH). The aim is to substantiate the necessity and importance of specialized care for the treatment of severely injured hand surgery patients and to demonstrate that patient flows need to be directed specifically to the appropriate centers. This provides important input for the further development of the healthcare landscape and supports the evidence-based design of the hospital reform.

## Materials and methods

This multicenter study is based on data from the HandTraumaRegister DGH (HTR DGH) of the German Society for Hand Surgery (Deutsche Gesellschaft für Handchirurgie, DGH), managed by the Academy for Trauma Surgery (Akademie der Unfallchirurgie GmbH, AUC) in collaboration with the DGH and other partner institutions. The HTR DGH is a national, multicenter registry that collects detailed information on hand injuries, treatment modalities, as well as injury-specific and demographic characteristics. Use and publication of the data for this study were approved by the HTR DGH following a two-stage review process under project ID 2023-005. The manuscript was also approved in accordance with the publication policy of the HTR DGH.

The inclusion criteria of the registry define the inclusion of cases with at least one finger or hand injury. The injury had to be treated surgically within 14 days. Both outpatient and inpatient cases were considered. Patient data entry into the central database is pseudonymized and performed via a web-based application.

For this analysis, data from the years 2018 (inception of the HTR DGH) through 2023 were used. The initial population comprised 27,607 completed patient cases from 55 participating hospitals. After exclusion of patients without documented finger injuries or patients with burns (*n* = 10,881), the final study population consisted of 16,726 cases from 54 hospitals. Hospitals were divided into three groups: a total of 13 centers were identified via their official websites as non-replantation centers. The remaining 41 centers, which publicly offer replantation services, were categorized as replantation centers. Among these, a subgroup held certification from the Federation of European Societies for Surgery of the Hand (FESSH). A total of 20 participating hospitals in the HTR DGH were FESSH-accredited.

In addition to general demographic variables (age, sex, handedness, insurance status), specific injury patterns (amputations, tendon injuries, fractures, nerve or arteria injuries), treatment types, and time-related parameters (e.g., time from admission to surgery, time from incision to closure) were analyzed. Documentation and data collection within the HTR DGH were conducted prospectively. Cases with unknown or missing data were excluded from the analysis of the respective variables.

All statistical analyses were performed using the latest version of R (version 4.3.2). For metric variables, the mean with standard deviation, and median with interquartile range (IQR) were calculated. Categorical variables were presented as absolute and relative frequencies. Group comparisons of two groups (replantation vs. non-replantation) were conducted using Student’s t test or Wilcoxon test, as appropriate. Group comparisons of three groups (replantation with FESSH vs. replantation without FESSH vs. non-replantation) were conducted using one-way ANOVA for normally distributed data or the Kruskal–Wallis test for non-normally distributed data. Associations between categorical variables for all group comparisons were analyzed using Pearson’s Chi-square test.

To examine the impact of hospital type on incision-to-closure time (outcome parameter in minutes), a multivariate linear regression model was employed. The effect of hospital type was included as the explanatory variable, with non-replantation centers defined as the reference category. Regression models were adjusted for age, gender, number of injured fingers and type of care.

Results are reported as exponential coefficients (exp(β)) with 95% confidence intervals (95% CI). Values of exp(β) > 1 indicate longer incision-to-closure times compared to the reference group, while values of exp(β) < 1 indicate shorter times. Statistical significance was determined based on the p-value, with *p* < 0.05 considered statistically significant.

## Results

A total of 16,726 patients were included in this study. Overall, 75.79% of the patients were male and 24.21% were female. In 26 cases, the gender was not recorded.

The proportion of right-handed individuals in the study population was 91.6%, while 7.4% were left-handed. Ambidextrous individuals accounted for 1% of the population. Regarding insurance status, 32.17% of cases were occupational accidents. In total, 8% of patients had private insurance, while the remaining 59.83% were covered by statutory health insurance.

Soft tissue injuries of the phalanges without fracture involvement or amputation injuries accounted for 46.57% of all cases. In 73.9% of cases, a single finger was injured. Injuries involving two fingers were present in 16.99% of cases, while more than two injured fingers were observed in 9.07% of cases. All fingers were injured in less than 1% of cases (0.44%).

### Door-to-skin-incision time

**Fig. 2:**
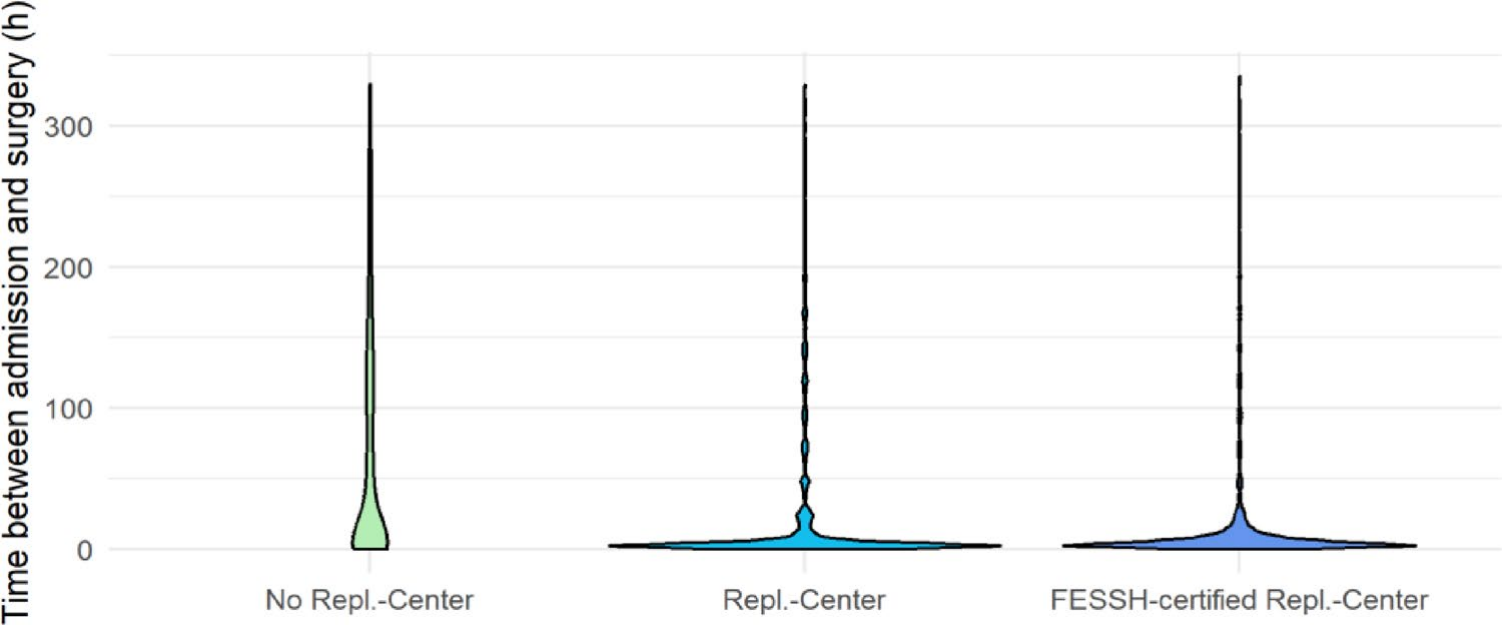
Door-to-skin-incision time by center type

The analysis of the time interval between initial consultation and the start of surgery revealed significant differences between the various hospital types. Non-replantation centers exhibited the longest door-to-skin-incision time, with a median of 13.23 h (IQR 2.78 h; 115.88 h). Replantation centers showed significantly shorter times, with a median of 3.75 h (IQR 2.17 h; 11.27 h). FESSH-certified replantation centers had the second shortest median door-to-skin-incision time at 4.83 h (IQR 2.62 h; 10.28 h) (Fig. 2).

The type of surgical treatment also differed significantly between the hospital categories (*p* < 0.001). Primary treatment was performed in 52.28% of cases in non-replantation centers, whereas this proportion was markedly higher in replantation centers (85.69%) and FESSH-certified replantation centers (79.03%). Consequently, secondary procedures were significantly more frequent in non-replantation centers (47.72%) compared to replantation centers (14.31%) and FESSH centers (20.97%). Definitive treatment was the most common therapeutic approach across all center types, defined as the final management of the injury. While 94.67% of cases in non-replantation centers underwent definitive treatment, this proportion was slightly lower in replantation centers (90.88%) and FESSH centers (87.27%). Temporary treatments were more frequent in replantation centers (6.02%) and especially in FESSH centers (8.06%) than in non-replantation centers (2.06%).

**Fig. 3:**
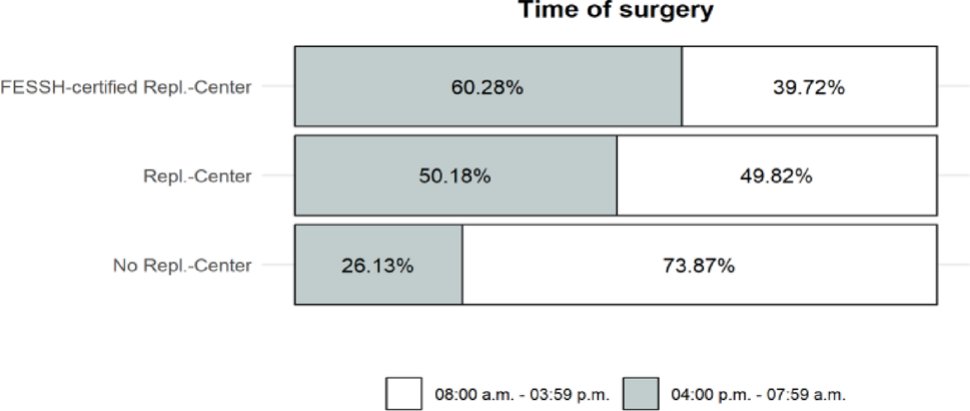
Distribution of surgery times across center types

Replantations or revascularizations were performed in 3.96% of cases overall, with the highest rate observed in FESSH centers (4.64%). The timing of surgery also varied significantly between center types (*p* < 0.001). In non-replantation centers, the majority of procedures (73.87%) were performed during regular working hours (8 a.m. to 3.59 p.m.), whereas surgeries in replantation centers (50.18%) and particularly in FESSH centers (60.28%) more frequently occurred during off-hours, between 4 p.m. and 8 a.m. (Fig. 3).

### Comparison of incision-to-suture time for different injury patterns depending on center type

**Fig. 4:**
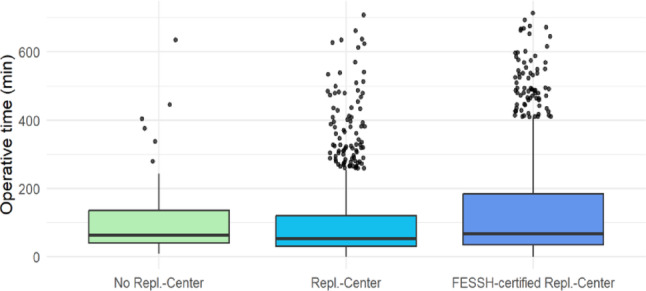
Operative Time of Amputation injuries across center types

A significant difference was observed in incision-to-suture time for amputation injuries between the various surgical centers (*p* < 0.001) (Table 1). FESSH-certified replantation centers exhibited the longest average incision-to-suture time at 129.7 min (range: 35.0 min; 184.5 min), followed by non-replantation centers with an average of 104.2 min (40.5 min; 135.75 min), and replantation centers without FESSH certification with an average of 98.6 min (30.0 min; 120.0 min) (Fig. 4).

**Table 1:** Incision-to-suture time across center types for different injury patterns (univariate analysis)

Injury pattern	Center type	Incision-to-suture time (mean/min or median)	Range / IQR	*p*-value
Amputation injuries	FESSH-certified replantation centers	129.7 min	35.0–184.5	< 0.001*
	Non-replantation centers	104.2 min	40.5–135.8	
	Replantation centers (non-FESSH)	98.6 min	30.0–120.0	
Flexor tendon injury (thumb)	FESSH-certified / replantation centers	118.9 min	51.0–164.5	0.045*
	Non-replantation centers	92.8 min	40.0–97.5	
Flexor tendon transection (fingers 2–5)	Replantation centers	115.6 min	49.5–159.0	n.s.
	Non-replantation centers	110.0–118.2 min	52.0–146.0	

This table summarizes the unadjusted comparison of incision-to-suture times across different injury patterns and center types. Data are presented as mean values with range or IQR as available. Differences between groups were assessed using appropriate statistical tests as indicated.

*Statistical significance is indicated by *p* < 0.05

**Fig. 5:**
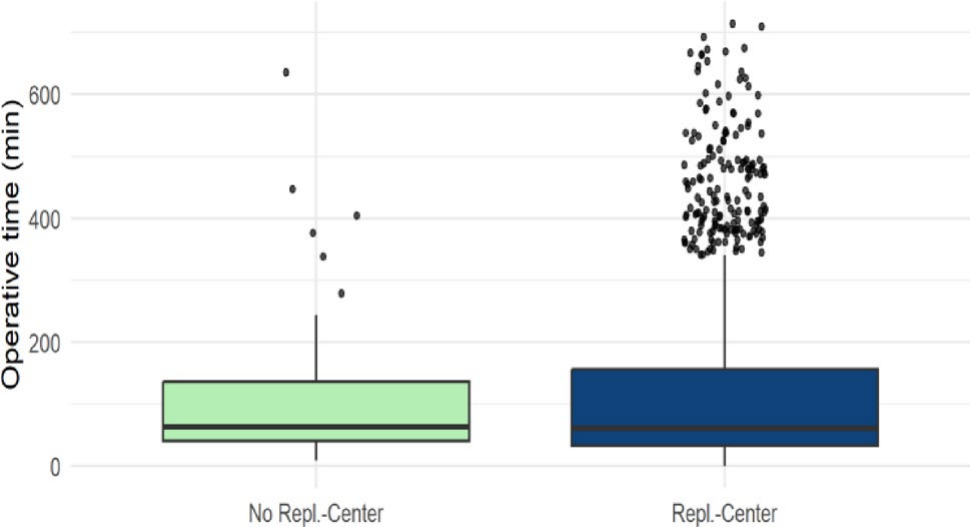
Comparison of Operative time between Replantation and No-replantation centers regarding amputation injuries

**Fig. 6:**
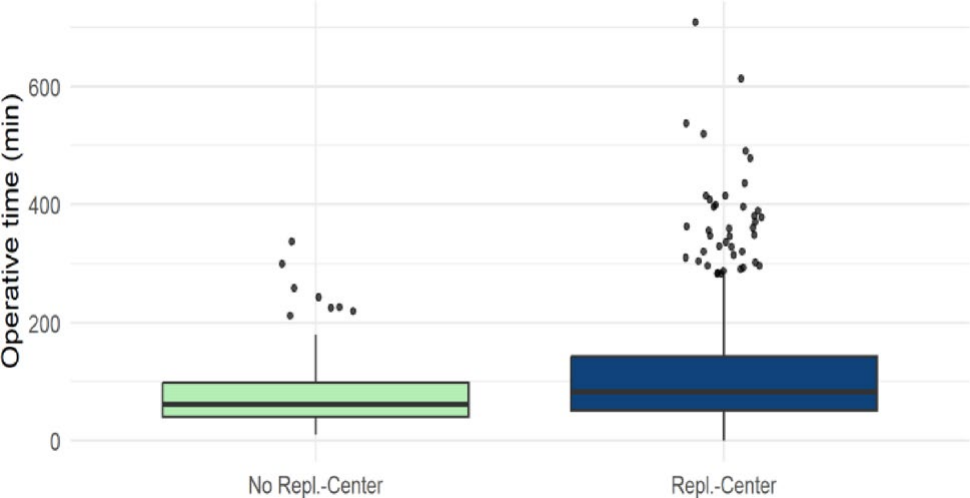
Operative time of flexor thumb injuries

**Fig. 7:**
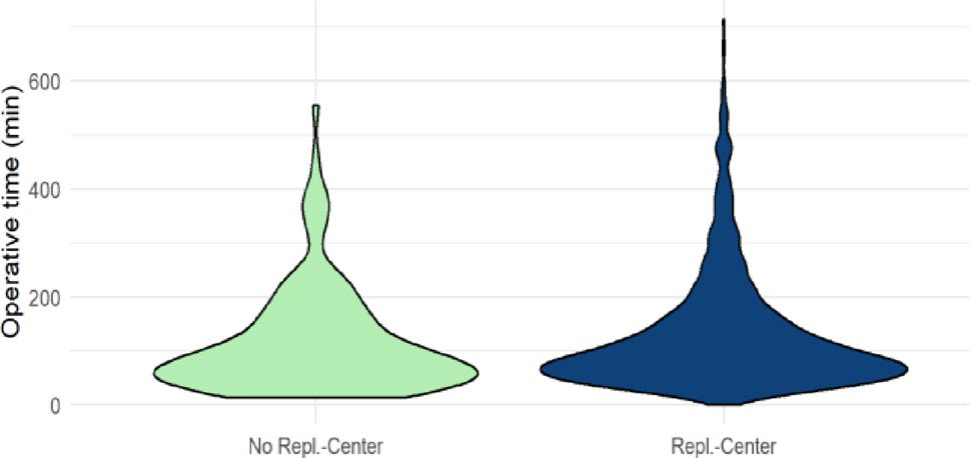
Operative Time of Flexor tendon injuries of the digits 2–5

Flexor tendon injuries of the thumb are rare, occurring in less than 4% of recorded finger injuries. In cases of flexor tendon injury, 97.4% involved complete transection. The type of center—replantation center with or without certification, and non-replantation center—had a significant impact on incision-to-suture time (118.9 min [51.0 min; 164.5 min] vs. 110 min [52.0 min; 126.5 min] vs. 92.8 min [40.0 min; 97.5 min], *p* = 0.045) (Fig. 6). A similar pattern was observed for flexor tendon transections of fingers 2–5. Here, too, incision-to-suture times differed significantly depending on the surgical center, although the difference between non-replantation centers and replantation centers without certification was not statistically relevant (115.6 min [49.5 min; 159.0 min] vs. 118.2 min [58.0 min; 146.0 min]) (Fig. 7).

For extensor tendon injuries, a similar trend was seen in the overall group, with most injuries being complete transections. In cases of extensor tendon transection of fingers 2–5, the type of center had a significant impact on incision-to-suture time (*p* < 0.001), with FESSH-certified replantation centers showing the longest average incision-to-suture time, followed by replantation centers without certification, and non-replantation centers (Table 1).

### Comparison of incision-to-suture time across different injury patterns between replantation centers with and without FESSH certification and non-replantation centers

The comparison of incision-to-suture times between replantation centers and non-replantation centers revealed significant differences. Considering all amputation injuries, the average incision-to-suture time in replantation centers was significantly longer at 68.1 min [IQR 26.0 min; 78.0 min] compared to non-replantation centers, which averaged 57.2 min [IQR 25.0 min; 66.0 min] (*p* < 0.001) (Table 2). Subgroup analysis of amputation injuries showed greater variability, with a tendency toward longer incision-to-suture times in replantation centers.

**Table 2:** Comparison of incision-to-suture times between replantation and non-replantation centers

Injury pattern	Replantation centers (mean/IQR)	Non-replantation centers (mean/IQR)	*p*-value
Amputation injuries	68.1 min (26.0–78.0)	57.2 min (25.0–66.0)	< 0.001*
Flexor tendon (thumb)	115.2 min (51.0–143.0)	92.8 min (40.0–97.5)	0.02*
Extensor tendon (thumb)	71.5 min (30.0–82.0)	55.3 min (27.5–69.5)	0.139
Extensor tendon (fingers 2–5)	87.3 min (31.0–110.0)	68.9 min (26.0–80.0)	< 0.001*

This table presents a subgroup analysis comparing incision-to-suture times between replantation centers and non-replantation centers for different injury patterns. Values are reported as mean with IQR

* Statistical significance is indicated by *p* < 0.05

For flexor tendon injuries of the thumb, the type of center (replantation vs. non-replantation) significantly influenced incision-to-suture time. In both FESSH-certified and non-certified replantation centers, the mean time was significantly longer at 115.2 min [IQR 51.0 min; 143.0 min] compared to 92.8 min [IQR 40.0 min; 97.5 min] in non-replantation centers (*p* = 0.02).

Similarly, for extensor tendon injuries, replantation centers demonstrated significantly longer incision-to-suture times than non-replantation centers. This was observed for extensor tendon injuries of the thumb (71.5 min [IQR 30.0 min; 82.0 min] vs. 55.3 min [IQR 27.5 min; 69.5 min], *p* = 0.139) and for extensor tendon injuries of fingers 2–5 (87.3 min [IQR 31.0 min; 110.0 min] vs. 68.9 min [IQR 26.0 min; 80.0 min], *p* < 0.001).

Overall, the results indicate that replantation centers generally have longer incision-to-closure times than non-replantation centers.

### Multivariate comparison of incision-to-suture time for different injury patterns between replantation centers with and without FESSH certification and non-replantation centers

In the overall group of amputation injuries, the type of center—replantation center with or without certification—did not have a significant impact on incision-to-suture time compared to non-replantation centers (exp() = 1.05, 95% CI: 0.87–1.26, *p* = 0.619) (Table 3).

**Table 3:** Multivariate analysis of incision-to-suture time: replantation centers vs. non-replantation centers

Injury pattern	exp(β)	95% CI	*p*-value
Amputation injuries (overall)	1.05	0.87–1.26	0.619
Flexor tendon thumb	1.11	0.90–1.35	0.329
Flexor tendon fingers 2–4	1.11	0.98–1.26	0.094
Extensor tendon thumb	1.09	0.88–1.38	0.42
Extensor tendon fingers 2–5	1.07	0.96–1.21	0.194

This table shows adjusted effects of center type on incision-to-suture time for different injury patterns using multivariate regression models. Results are expressed as exp(β) with 95% confidence intervals

*Statistical significance is indicated by *p* < 0.05

Similarly, in the subgroups of amputation injuries involving fingers DI to DV, no significant effect was observed. The exp() values ranged from 0.83 to 1.15, with all p-values being non-significant (DI: *p* = 0.813, DII: *p* = 0.137, DIII: *p* = 0.917, DIV: *p* = 0.309, DV: *p* = 0.463).

For flexor tendon transection of the thumb, the type of center (replantation center) also showed no significant influence on incision-to-suture time compared to non-replantation centers (exp() = 1.11, 95% CI: 0.9–1.35, *p* = 0.329). For flexor tendon transections of fingers 2–4, no significant effect was detected, although there was a trend toward slightly prolonged incision-to-suture times (exp() = 1.11, 95% CI: 0.98–1.26, *p* = 0.094). For extensor tendon transections of the thumb, the type of center had no significant effect on incision-to-suture time (exp() = 1.09, 95% CI: 0.88–1.38, *p* = 0.42). A similar result was found for extensor tendon transections of fingers 2–5, where no significant effect was observed (exp() = 1.07, 95% CI: 0.96–1.21, *p* = 0.194).

### Multivariate comparison of incision-to-suture time for different injury patterns between FESSH-certified and non-FESSH-certified replantation centers

Overall, FESSH-certified replantation centers showed a significantly longer incision-to-suture time for amputation injuries compared to non-FESSH-certified replantation centers. This is reflected by an exp() value of 1.25 (95% CI: 1.16–1.35, *p* < 0.001) in the multivariate analysis, corresponding to an average increase in incision-to-suture time of 25% in FESSH-certified centers (Table 4).

**Table 4:** Multivariate analysis of incision-to-suture time: FESSH-certified vs. non-FESSH-certified replantation centers

Injury pattern	exp(β)	95% CI	*p*-value
Amputation injuries (overall)	1.25	1.16–1.35	< 0.001*
Amputation DV	1.14	0.95–1.38	0.149
Flexor tendon thumb	1.05	0.94–1.17	0.381
Flexor tendon fingers 2–4	1.09	1.02–1.17	0.008*
Extensor tendon thumb	1.01	0.89–1.15	0.871
Extensor tendon fingers 2–5	1.05	0.98–1.13	0.149

This table presents adjusted regression results comparing FESSH-certified and non-certified replantation centers across different injury patterns. Data are shown as exp(β) with 95% confidence intervals

*Statistical significance is indicated by *p* < 0.05

For amputations of fingers DI to DIV, a significant effect of center type was also observed, with exp() values ranging from 1.27 to 1.34 (*p* < 0.001), indicating systematically longer incision-to-suture times in FESSH-certified centers. For amputations of finger DV, however, no significant effect was found (exp() = 1.14, 95% CI: 0.95–1.38, *p* = 0.149).

For flexor tendon transection of the thumb, certification status had no significant effect (exp() = 1.05, 95% CI: 0.94–1.17, *p* = 0.381). For flexor tendon transections of fingers 2–4, a significant effect was detected, with FESSH certification increasing treatment duration by an average of 9% (exp() = 1.09, 95% CI: 1.02–1.17, *p* = 0.008). For extensor tendon transections of the thumb, center type had no significant effect on incision-to-suture time (exp() = 1.01, 95% CI: 0.89–1.15, *p* = 0.871). A similar result was observed for extensor tendon transections of fingers 2–5, where no significant effect was found (exp() = 1.05, 95% CI: 0.98–1.13, *p* = 0.149).

## Discussion

### Door-to-skin-incision time

The longer delays observed in non-replantation centers may be attributed to several factors. These might include a lower prioritization of hand injuries, limited availability of specialized surgeons, and organizational challenges such as restricted operating room capacity and prolonged decision-making processes.

The significantly shorter door-to-skin-incision-time in replantation centers suggests more efficient OR scheduling, faster diagnostics and more rapid decision-making. Furthermore, the significantly reduced door-to-incision time highlights the continuous presence of specialized hand surgery personnel in replantation centers, both with and without FESSH certification. Notably, FESSH-certified centers exhibit the shortest delays, which may reflect higher specialization levels and standardized treatment protocols. However, despite statistical significance, the median difference of 1.08 h between FESSH-certified and non-certified replantation centers, coupled with considerable variability, is likely clinically negligible.

Another important observation is the markedly higher rate of definitive treatment in replantation centers. Definitive treatment typically refers to the final surgical management performed during the initial operation. Temporary procedures were more common in replantation centers (6.02%) and especially in FESSH-certified centers (8.06%) compared to non-replantation centers (2.06%), suggesting a more nuanced and tailored therapeutic approach in specialized facilities.

Since replantation centers, irrespective of FESSH certification, required fewer secondary surgeries, this may contribute to a reduction in overall healthcare costs by avoiding additional operative procedures and hospital stays. Simultaneously, patients face a reduced risk of repeated anesthesia and further surgical complications, potentially improving patient safety and accelerating rehabilitation. Ultimately, faster definitive treatment of complex hand injuries with fewer follow-up surgeries could shorten hospital length of stay, which might translate into healthcare cost savings.

Since diagnosis-related groups (DRG) are not captured in the HTR, potential economic effects cannot be directly demonstrated within this dataset. Furthermore, length of hospital stay was not available for all patients, making a reliable comparison of inpatient stay durations between centers impossible. Our findings emphasize the importance of specialized treatment centers for the timely and appropriate surgical management of finger injuries; however, conclusions regarding potential cost or healthcare system benefits must be interpreted with caution in light of the missing economic data. For certain isolated injuries (e.g., nerve lesions), it is known that early intervention is associated with better functional outcomes [9]. Therefore, it is conceivable that early definitive treatment in replantation centers may improve treatment quality—however, robust conclusions regarding health policy or economic benefits require additional specifically collected DRG, cost, or length-of-stay data.

Replantations or revascularizations were performed in 3.96% of cases overall, with the highest proportion in FESSH-certified centers at 4.64%. This highlights the role of these centers in the microsurgical reconstruction of complex finger injuries.

### Incision-to-suture time

The longer incision-to-suture times observed in FESSH-certified replantation centers might suggest a higher complexity of cases treated in these specialized facilities. Both replantation centers and FESSH-certified replantation centers showed significantly longer operative times for surgical management compared to non-replantation centers. The significantly longer incision-to-suture times in FESSH-certified centers, compared to non-replantation centers, can likely be explained by higher case complexity. These specialized centers more frequently manage complex injury patterns, such as proximal amputations, multifragmentary fractures, or combined vascular, nerve, and tendon injuries that require more elaborate microsurgical reconstruction.

Additionally, structural factors such as the involvement of multiple specialties, a strong focus on surgical training with participation of residents, and comprehensive intraoperative documentation for quality assurance may contribute to longer operative times. A possible referral bias, in which especially complex or secondary cases are transferred to specialized centers, is also likely to further influence differences in operative duration.

### Analysis of care structures

Analysis of care structures shows that for patients with occupational accident insurance (BG cases), specialized treatment protocols are already established through the so-called “Schwerstverletzungsartenverfahren” (SAV) [10]. This protocol, initiated by the statutory accident insurance institutions, aims for targeted treatment of severe injuries in specially certified centers with the necessary expertise and infrastructure [11].

In the studied cohort, 32.17% of patients had occupational accident insurance. These patients were treated significantly more often in specialized centers than those with other types of insurance (*p* < 0.001). Especially in replantation and FESSH-certified centers, there was a higher concentration of BG cases, who benefit from the specific requirements for rapid and complex care.

Positive experiences with the SAV protocol for BG cases demonstrate that structured and targeted centralization in specialized facilities can make a decisive contribution to improving care for severely injured patients. These findings can serve as a model for further development of the healthcare landscape within the framework of hospital reform, enabling targeted referral of non-BG patients to appropriate centers as well.

### Timing of surgery

The timing of surgery also differed significantly between the various types of centers (*p* < 0.001). While the majority of procedures in non-replantation centers (73.87%) were performed within regular working hours (8 a.m. to 3.59 p.m.), operations in replantation centers (50.18%) and especially in FESSH-certified centers (60.28%) were performed much more frequently outside this time frame. This pattern suggests that specialized centers have greater readiness and structural capacity to provide emergency care outside of regular working hours. The increased number of procedures performed in the evening and at night likely reflects both the higher complexity and urgency of cases treated in these facilities, as well as their organizational focus on time-critical management of severe hand injuries and replantations. The presence of a dedicated replantation service also plays a role here. This criterion, which was used to classify centers as “replantation center” or “non-replantation center,” is, in our opinion, reflected in the operative times after 4 p.m. However, statements about the quality of care after 4 p.m. cannot be made based on the available data from the HTR DGH.

### Limitations

This retrospective analysis focuses exclusively on the time intervals between hospital admission and the start of surgery, as well as the incision-to-suture time, serving as temporal indicators of care. Whether the procedure was performed by a microsurgically experienced specialist or a senior surgeon is not documented in the HandTraumaRegister (HTR DGH). While the findings provide valuable insights into organizational and structural differences between non-replantation centers, replantation centers, and FESSH-certified replantation centers, the specific surgical techniques employed and their quality are not addressed, representing a major limitation of this study.

Furthermore, diagnosis-related group (DRG) codes and data on hospital length of stay are not comprehensively captured. Length of stay has only been documented in recent years and was unavailable during the early phase of the HTR DGH, making an analysis of this parameter currently impossible. However, the authors consider such data essential for a thorough economic evaluation of these injuries, particularly in the context of ongoing healthcare reforms in Germany. In addition, no clinical follow-up examinations were performed to correlate the recorded time intervals with functional outcomes. Similarly, no standardized scales, outcome scores, or quality-of-life questionnaires were collected within the HTR DGH, further limiting the assessment of patient-centered results.

### Differences in types of surgical procedures

The findings show that complex multiple injuries are less frequently treated in non-replantation centers compared to specialized centers. Additionally, the HTR DGH does not specify the level of training or experience of the surgeon performing the procedure. It is conceivable that surgical training cases under supervision might contribute to prolonged incision-to-suture times. The observed differences in incision-to-suture times between replantation and non-replantation centers cannot be conclusively explained based on the available HTR DGH data. It also cannot be demonstrated that simpler or inferior treatment occurs in non-replantation centers. In our opinion, factors such as training procedures may explain median incision-to-suture time differences of approximately ± 60 min.

Studies have shown that complex procedures such as replantations, which are predominantly performed in specialized centers, are associated with better functional and long-term results [7]. The sole focus on temporal parameters without considering the surgical techniques applied limits the interpretability of this analysis. A detailed investigation of the surgical methods employed and their outcomes is necessary to fully capture qualitative differences between centers. The type of surgical treatment is not recorded in detail within the HTR. While the registry captures temporal parameters and basic clinical information, it does not provide a differentiated documentation of the specific operative procedures or surgical techniques performed. As a result, the exact nature of the surgical management in individual cases cannot be clearly reconstructed. This lack of granularity represents a relevant limitation in the interpretation of the findings, particularly with regard to potential differences in treatment strategies between participating centers.

This limitation emphasizes the need for further studies that consider not only organizational aspects but also the surgical techniques applied and their impact on patient outcomes.

In addition, potential selection bias inherent to registry-based analyses should be acknowledged. As participation in the Hand Trauma Register (HTR DGH) is voluntary and dependent on contributing centers, the dataset may not fully represent all treated cases or institutions. Furthermore, registry-based data are inherently limited by the predefined structure of data entry, which may restrict the granularity of clinical information and introduce reporting bias. These general registry-related limitations should be considered when interpreting the findings of this study.

## Data Availability

The data used in this study were obtained from the HandTraumaRegister of the German Society for Hand Surgery (HTR DGH). The datasets are anonymized and are not publicly available due to data protection regulations and registry governance policies. However, data may be made available from the corresponding registry upon reasonable request and with permission of the HTR DGH.

## Abbreviations

BG: Berufsgenossenschaft” Professional Association (German Social Accident Insurance)
DRG: Diagnosis Related Groups
FESSH: Federation of European Societies for Surgery of the Hand
DGH: German Society for Hand Surgery
HTR DGH: HandTraumaRegister of the German Society for Hand Surgery
min: Minutes
NR: Non Replantation Center
QALY: Quality–Adjusted Life Years
SAV: “Schwerstverletzungsartenverfahren” Severe Injury Type Procedure
AUC: Academy of Trauma Surgery GmbH
